# The association between income inequality and adult mental health at the subnational level—a systematic review

**DOI:** 10.1007/s00127-021-02159-w

**Published:** 2021-08-13

**Authors:** Marc S. Tibber, Fahreen Walji, James B. Kirkbride, Vyv Huddy

**Affiliations:** 1 Department of Clinical, Educational and Health Psychology, UCL, London, UK; 2 PsyLife Group, Division of Psychiatry, UCL, London, UK; 3 Clinical and Applied Psychology Unit, Department of Psychology, University of Sheffield, Sheffield, UK

**Keywords:** Inequality, Deprivation, Poverty, Social determinants, Mental health

## Abstract

**Purpose:**

A systematic review was undertaken to determine whether research supports: (i) an association between income inequality and adult mental health when measured at the subnational level, and if so, (ii) in a way that supports the Income Inequality Hypothesis (i.e. between *higher* inequality and *poorer* mental health) or the Mixed Neighbourhood Hypothesis (*higher* inequality and *better* mental health).

**Methods:**

Systematic searches of PsycINFO, Medline and Web of Science databases were undertaken from database inception to September 2020. Included studies appeared in English-language, peer-reviewed journals and incorporated measure/s of *objective* income inequality and adult mental illness. Papers were excluded if they focused on *highly* specialised population samples. Study quality was assessed using a custom-developed tool and data synthesised using the vote-count method.

**Results:**

Forty-two studies met criteria for inclusion representing nearly eight million participants and more than 110,000 geographical units. Of these, 54.76% supported the Income Inequality Hypothesis and 11.9% supported the Mixed Neighbourhood Hypothesis. This held for highest quality studies and after controlling for absolute deprivation. The results were consistent across mental health conditions, size of geographical units, and held for low/middle and high income countries.

**Conclusions:**

A number of limitations in the literature were identified, including a lack of appropriate (multi-level) analyses and modelling of relevant confounders (deprivation) in many studies. Nonetheless, the findings suggest that area-level income inequality is associated with poorer mental health, and provides support for the introduction of social, economic and public health policies that ameliorate the deleterious effects of income inequality.

**Clinical registration number:**

PROSPERO 2020 CRD42020181507.

**Supplementary Information:**

The online version contains supplementary material available at 10.1007/s00127-021-02159-w.

## Introduction

Mental disorders are the leading cause of years lived with disability worldwide [1]. Whilst this has led to calls for greater investment in psychological therapies [2], of which the UK’s improving access to psychological therapies (IAPT) scheme is a prime example [3], such an approach, which (arguably) locates the problem *as well as the solution* in the individual, has had its detractors. Thus, many have proposed that such an approach fails to take into consideration the socioeconomic contexts in which mental illness, and distress more generally, occurs, and consequently, removes the onus on governments for broader social and economic reform [4–6].

With respect to the existing evidence-base, the association between income and health is well established [7]. For example, life expectancy increases as a function of gross national product (GNP), though the effects typically saturate at higher levels of GNP [8, 9]. Whilst there are less data on mental health, there is evidence to suggest that mental health and wellbeing show a similar asymptotic relationship with GNP between nations [10–12]. One interpretation of these findings is that in poorer countries, income—and specifically a minimum level of income—is directly linked to health outcomes, since poverty limits access to basic needs such as food and clean water, i.e. poverty is associated with *material* deprivation. In contrast, in countries above a certain threshold of wealth, these factors become less important for a larger majority of the population, as basic needs are satisfied.

Looking at data *within* a country, e.g. comparisons across states or counties, income similarly predicts physical [13] and mental health outcomes [14–16], but unlike cross-national comparisons, the effects do not seemingly saturate at higher incomes. One explanation is that whilst income is an index of access to basic amenities in comparisons *across* countries, *within* a country income becomes an indicator of social position or socioeconomic status (SES). This is important, because a large body of research has shown that SES is inversely related to unhealthy behaviours such as smoking, physical inactivity and unhealthy eating [17].

According to the Income Inequality Hypothesis (IIH) [18], it is not just socioeconomic position per se that affects health, but socioeconomic position relative to others around you, namely inequality, i.e. the *variance* in incomes (or some related index of poverty or wealth) within a defined region. To characterise levels of objective inequality within a region several measures have been developed, including decile ratios, the Robin Hood index, and Gini coefficient, all of which correlate highly with one another [19]. The Gini coefficient is the most commonly used, and describes the extent to which the distribution of incomes in a region deviates from perfect equality, with high scores indicating high variance. In Wilkinson and Pickett’s book, ‘The Spirit Level’ [11], the authors popularised the IIH, describing how the Gini coefficient positively predicts an aggregate index of health and social problems, as well as related indices such as obesity [20], life expectancy [21], incarceration, homicide rates, education and levels of childhood conflict [22, 23], both in cross-country comparisons as well as subnational comparisons between US states. Whilst a number of criticisms have been raised against Wilkinson and colleagues’ analyses [24–26], the principle finding of an association between higher inequality and poorer *physical* health and social outcomes, though small, has since been confirmed [27–30].

With respect to the possible mechanisms underlying the association between income inequality and health, three main theories have been proposed [31, 32]. According to the Social Capital Hypothesis (SCH) when individuals or groups of individuals differ greatly in their incomes (i.e. conditions of *high* inequality), they are less likely to trust one another, or to interact and form cohesive social networks [33], which may be inherently stressogenic [34]. Such conditions are also less likely to engender acts of reciprocity and practical support [35]. In contrast, the Status Anxiety Hypothesis (SAH) proposes that income inequality leads to greater social comparison between the rich and poor, which may also be stressful and detrimental to health [36, 37]. Finally, the Neomaterialist Hypothesis (NMH), posits that when levels of inequality are high, less investment is made into public infrastructure and welfare services [38–40], e.g. gyms, parks and hospitals, which in turn, leads to poorer health outcomes [41].

Others have proposed an association between health and inequality that runs contrary to the IIH, i.e. an association between higher inequality and *better* health. According to the Mixed Neighbourhood hypothesis (MNH) [42–44], whilst neighbourhoods of *homogeneous* poverty, i.e. areas of high deprivation but *low* inequality, may become mired by a lack of social opportunities and cultures of crime, substance use and joblessness, the MNH proposes that these effects can be ameliorated by integration with individuals of a higher SES, i.e. areas of high deprivation but *high* inequality also. On a purely pragmatic level, poorer members of the community may benefit from the increased investment in local infrastructure and resources that such heterogeneity brings. In some countries this has led to the adoption of mixed-income housing development schemes, e.g. the HOPE VI project [45], although this is a highly controversial approach, which some have argued is founded on insufficient evidence [46–48].

Despite growing interest, there has been less research into the association between inequality and *mental* health than there has into the association with *physical* health [49]. Nonetheless, several systematic reviews of relevance have been undertaken. Burns and colleagues [50] undertook a systematic review of schizophrenia, and found that across data from 26 countries, there was a higher incidence rate of the condition in higher income countries (β = 1.02; *Z* = 2.28; *p* = 0.02; 95% CI = 1.00, 1.03). In a systematic review and meta-analysis of depression [51], from 26 papers (of which 12 were included in the meta-analysis), the authors reported a greater risk of depression in populations with higher inequality (RR = 1.19, 95% CI = 1.07–1.31).

Only one review paper to date [52], however, has attempted to synthesise the literature on the association between inequality and mental health *across* different presentations. In their paper, the authors undertook a systematic review of 27 papers and a meta-analysis of nine studies, and concluded that there was a weak association between higher income inequality and *any* mental health difficulty (pooled Cohen’s *d* = 0.06, 95% CI = 0.01–0.11). However, in defining their search terms they included only broad definitions of mental health problems rather than specific diagnostic categories. Consequently, a number of studies of relevance may have been missed, and biases may have been introduced with respect to study selection. In addition, they did not assess the impact on their findings of including only studies that had controlled for *absolute* deprivation. However, without controlling for *absolute* deprivation, any reported effects of inequality may be driven by this factor rather than inequality per se [53, 54].

To address these limitations, we undertook a systematic review of the association between inequality and mental health using a comprehensive set of search terms that included specific as well as broad definitions of mental health (and inequality), thereby ensuring good coverage. To disentangle the potential confounding effects of *absolute* deprivation in any studies, we also explored the extent to which any documented patterns persisted in a subset of papers that controlled for deprivation at either the individual or area level (or both).

In addition, we explored a number of more specific predictions that have been made in relation to the IIH. First, that the association between inequality and health is not restricted to the poor, but is instead present in the rich also, i.e. the effect does not interact with *absolute* deprivation [11]. Second, that the effects of IIH do not hold across different geographical scales. Thus, in trying to make sense of the literature, Pickett and Wilkinson [55, 56] have proposed that the effects of inequality become weaker—or possibly do not even operate—at smaller scales, e.g. in comparisons between geographical areas below the level of US states, for example. Finally, we include only studies that describe analyses undertaken at the subnational level, e.g. comparisons across neighbourhoods or states rather than across countries, since first, as noted, socioeconomic processes may function differently in cross-national comparisons, and secondly, because this is the level at which mental health services are typically commissioned, designed and delivered, and political decisions are made.

## Methods

This review represents an update of an unpublished thesis [57] prospectively registered with PROSPERO before the search was updated (CRD42020181507) [58]. The study is reported according to PRISMA guidelines [59]. A meta-analytic approach was *not* adopted since aggregation of effect sizes is inappropriate when studies differ markedly with respect to sample characteristics, outcome variables, methodologies and analytic approaches [60–62]. Instead, we conducted a narrative review, searching for broad patterns of support for opposing hypotheses (the IIH and MNH) coupled with a vote-count approach [56, 63]. All studies were screened and coded independently by MT and FW. Findings were then reviewed together after each sequential step and any discrepancies discussed and resolved, with further input sought from JK where needed.

### Search strategy

Studies were identified using a search of PsycINFO, Medline and Web of Science databases from database inception to the 2nd September, 2020, with no restriction on studies that could be included within this temporal window. A comprehensive set of search terms were based on the two key concepts of ‘income inequality’ (11 terms) and ‘mental health’ (52 terms); see Supplementary Information 1.

### Screening and selection

**Fig. 1:**
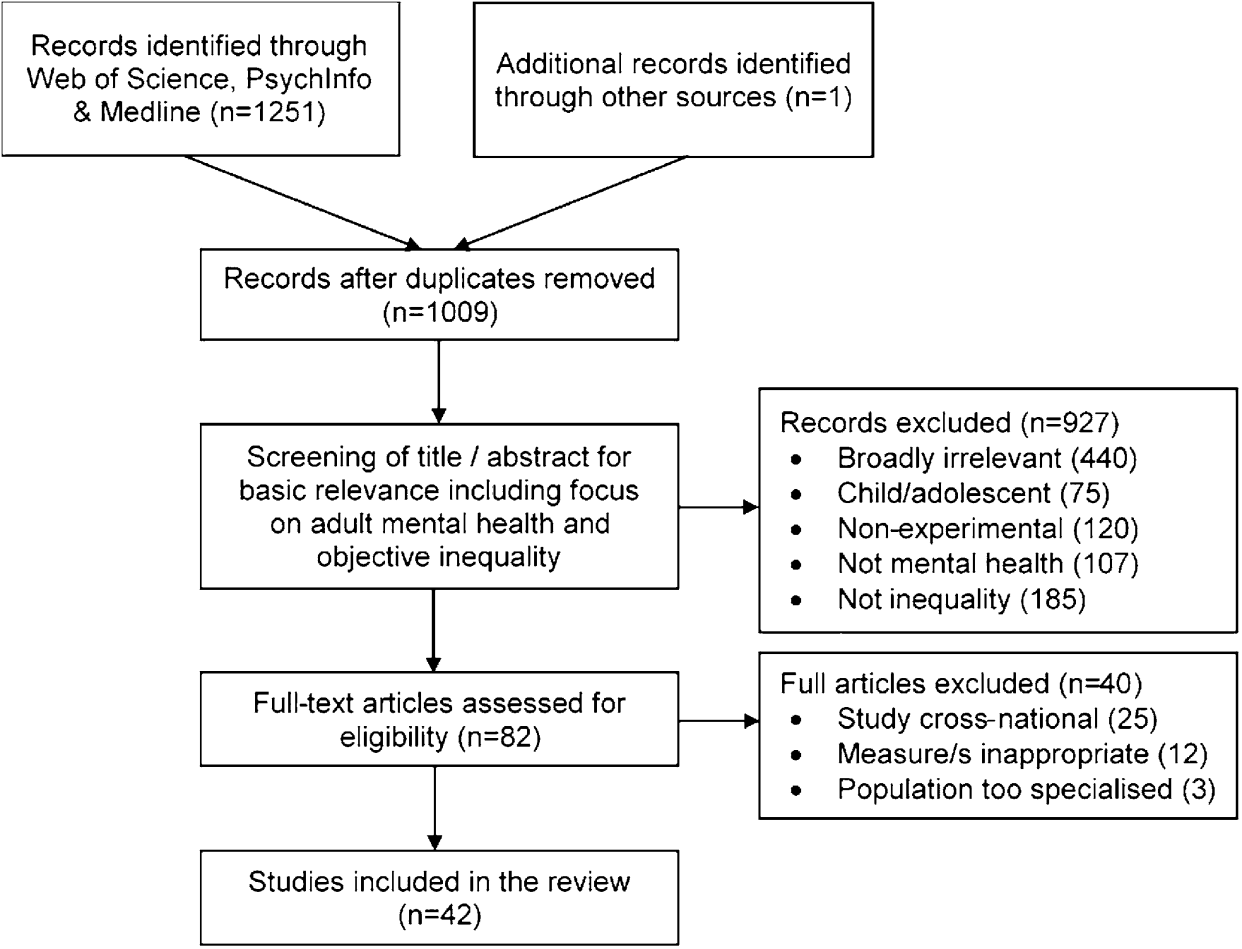
Study inclusion flow diagram. Flow diagram showing sequence by which studies were identified, screened and reviewed

All records were screened in two phases (see Fig. 1). First, the title and abstract were screened and methods section reviewed for basic relevance including a focus on mental health and objective inequality. Second, all remaining articles were read and relevant studies identified according to the following inclusion criteria: (i) included quantitative data; (ii) included a measure of mental illness incidence, prevalence or symptom severity, defined using a diagnostic tool, screening instrument or symptom scale; (iii) included an objective measure of income inequality, derived at the subnational level; (iv) focused on *adult* mental health (≥ 18 years); (v) written in English; and (vi) published in peer-reviewed journals. Studies were excluded: (i) if the measure of inequality was based on *subjective* inequality; (ii) if the focus was on life satisfaction, health-care use, neurodevelopmental disorders, learning disabilities, degenerative diseases or behaviour, e.g. suicide or substance use; (iv) if the sample population was based on a *highly* specialised population sample, e.g. HIV + prisoners [64].

### Data extraction

Remaining studies were coded for key measures to facilitate synthesis of findings and assessment of study quality (see Table 1). These included: the scale of the geographical region of interest, mean population size of the region of interest, data sample size (at individual and higher-order level), the type of analyses undertaken, predictors and covariates included in analyses, the significance of any findings at an alpha criterion level of 0.05, as well as an index of study quality (see Supplementary Information 2). Further information about studies is also presented in Supplementary Information 3. Where data were *not* specified in a given study, this information was sought from original sources, e.g. government reports and national statistics, requested directly from the study’s authors, and where not available coded ‘NA’.

**Table 1:** Studies included in the review with key measures coded

Study	Data year	Country /focus of study	Area of interest	Area mean pop size	Inequality measure	MH variable	MH tool	*N*	Analyses	Lower level predictors	Higher level predictors	Conclusion	Qi
Ahern and Galea [67]	2000–2002 (2000)	US	Community district	125,000	GINI (income)	6-month prevalence of depression	National Women's Study (NWS) depression module	1355; 59	Multi-level logistic regression	Age, ethnicity, individual income	Income	Association between higher inequality and depression (low-income participants only) (β = 35.02, *p* < 0.01)	4
Adjaye-Gbewonyo et al. [105]	2008–2012 (2007, 2011)	South Africa	District council	1 million	Gini coefficient (income)	Symptoms of depression	CES-D-10	9664; 52	Multi-level linear regression	Age, gender, ethnicity, education level, household income, employment status, marital status, urban/rural location, receipt of any government grants	Mean household income, mean age, percent African, percent non-white, percent female, percentage of adults with no education, percentage of adults with completed further education, percentage of adults with higher education, percentage of adults unemployed, percentage of adults not economically active, percentage of rural households	No association (coefficient = 0.5, *p* > 0.05)	4
Bechtel et al. [95]	2001–2008	Australia	Neighbourhood, city and major statistical region	NA	GINI (income), Theil index, Atkinson Index	General mental health symptoms	MH component of the SF-36	67,305/40,753; 488 (major statistical region), NA (city), NA (neighbourhood)	Linear regression	Age, age-squared, number of dependents, region of birth, education, household income	None	No association (β = 1.16, *p* > 0.1)	2
Bisung et al. [106]	2009 (2010)	Ghana	Sub-metros in accra metropolitan area (and enumeration area)	19,588	GINI (“poverty”)	Dichotomised symptoms of depression	Single item self-report question	2814; 6 (sub-metro areas), 195 (enumeration areas)	Multi-level binary logistic regression	Age, marital status, number of children, length of stay, alcohol consumption, ever smoked, health insurance, level of education, wealth, community participation, tension with others. Employment status	Neighbourhood socioeconomic status, neighbourhood housing ownership, neighbourhood ethnic diversity,	No association (OR = 0.88, *p* > 0.05)	2
Bocoum et al. [79]	2002–2013	Canada	Regional county municipality	44,000	GINI (income)	Dichotomised self-reported presence of depression (proportion of sample self-reporting as depressed)	Single item self-report question	NA; 87	Binary logistic regression	None	Inequality, average disposable income, criminality rate, number of physicians	Income inequality was positively associated with depression at 3-year time lag only (proportion increase = 4.17, p < 0.01)	1
Boydell et al. [68]	1988–1997 (1991)	UK	Electoral ward	10,000	Median deviation from median deprivation	10-year incidence of psychosis	OCCPI	222; 15	Multi-level poisson regression	Age, sex, ethnicity	Deprivation, inequality, proportion ethnic minority	Association between higher inequality and FEP (most deprived wards only) (IRR = 3.79, p = 0.019)	2
Burns and Esterhuizen [69]	2005 (2001)	South Africa	Municipality	72,611	Ratio of mean income of highest to lowest decile earners	One-year incidence of first episode psychosis	Meeting DSM-IV criteria	160; 7	Partial correlation	Age, gender, ethnicity, employment status = included as covariates	Income, urbanicity	Association between higher inequality and FEP (*r* = 0.84, *p* = 0.036)	1
Burns et al. [80]	2008, 2010, 2012 (2005, 2006)	South Africa	District Municipality	925,000	P90/10 ratio	Dichotomised symptoms of depression	CES-D	15,505; 53	Multi-level binary logistic regression	Age, gender, education, employment status, ethnicity, marital status, assessment year, household income	None	Inequality was associated with higher likelihood of reporting depressive symptoms (beta = 0.04, p = 0.01), particularly in low-income households	3
Chen et al. [81]	2001–2003 (2000)	US	Census tract	4582	GINI (income)	Diagnosis of a mood, anxiety, alcohol or drug disorder	WMH-CIDI V3	13,775; 1394	Logistic regression	Age, gender, ethnicity, born in the US, education, household income, subjective socioeconomic status (relative to community and nation)	Neighbourhood affluence, neighbourhood race/ethnicity concentration, residential instability	Inequality predicts mood (OR = 1.07, *p* < 0.05) and anxiety disorders (OR = 1.08, *p* < 0.05), but not alcohol or drug disorders (except for black section of sample)	3
Chiavegatto Filho et al. [70]	2005–2007 (2010)	Brazil	Municipality, administrative region	287,884	GINI (income)	Prevalence of: (i) depression, (ii) anxiety, (iii) any MH disorder	WMH-CIDI	3542; 69	Bayesian multi-level logistic regression	Age, gender, income, education, marital status	None	Higher inequality associated with higher odds of any MH disorder (OR = 1.32, 1.24) and depression (OR = 1.76, 1.53); not significant for anxiety (OR = 1.25, 1.07)	3
Choi et al. [71]	2000–2010 (2000–2010)	US	County	193,750	GINI (income)	Self-rated health, depression symptoms & lifetime incidence of a psychiatric diagnosis	Self-rated health Status (SRH); CES-D; presence / absence of a psychiatric diagnosis	34,994 (propensity score matched); 2898	Logistic regression	Age, gender, race/ethnicity, marital status, education, wealth, income, years of living in/around current residence, household wealth decile, household income decile,	None	Higher inequality associated with higher odds of scoring highly on SRHS (OR = 1.12–1.17) and having had a psychiatric diagnosis (OR = 1.08–1.16) but not high scores on CES-D (OR = 1.05–1.09)	2
Cohen-Cline et al. [82]	2009–2013 (2010)	US	Census tract	4000	GINI (income)	Symptoms of depression	PHQ-2	3738 same-sex twin-pairs; > 1,300	Multi-level poisson regression	None	None	Inequality predicted depression symptoms between twin pairs (Rate Ratio = 1.78, CIs = 1.01–3.13) but did not predict variance within pairs	3
Dev and Kim [88]	2008–2014 (1990)	US	State	4.5 million	GINI (income)	Depression prevalence	CES-D-7	6997; 48	Multilevel logistic regression	Age, gender, ethnicity, marital status, education, net income	Median household income, race/ethnicity concentration, county-level social capital	Association between higher inequality and odds of depression (OR = 1.35, *p* < 0.05) nearly two decades later, which disappears after including county-level social capital	4
Ding et al. [87]	2006 (2006)	China	County, province	42 million (province), 460,000 (count)	GINI (income)	Schizophrenia prevalence	WHO Disability Assessment Schedule, Version II	1,909,205; 734 (county), 31 (provinces)	Multilevel logistic regression	Age, gender, urbanicity, education, marital status, household income, employment status	Median income	Association between higher inequality and risk of Schizophrenia at province (OR = 1.03, *p* < 0.0.5) but not county (OR = 0.99, *p* > 0.05) level. Former effect most pronounced in highest income quartile	4
Drukker et al. [94]	2000 (1998–2002)	Netherlands	Neighbourhood	3,389	Ratio of low to high incomes, house price standard deviation	General mental health symptoms	WHOQOL-BREF	1082; 36	Multi-level linear regression	Age, sex, occupation, education, welfare recipient, single-parent	Deprivation	No association (β = −0.03, *p* > 0.05)	3
Du et al. [83]	2010, 2014 (2010)	China	Province	45 million	GINI (household income)	Self-reported non-specific psychological distress	K6	22,112 (matched with GINI); 20	Multi-level linear regression	Age, gender, education, ethnicity, marital status, income, urban/rural residence, time 1 subjective wellbeing, time 1 psychological distress	None	Inequality predicted psychological distress (β = 1.04, *p* < 0.05), particularly in low-income families	3
Erdem et al. [107]	2012 (2012)	Netherlands	Neighbourhood and municipality	40,949 (municipalities), 2028 (neighbourhoods)	GINI (standardized disposable household income)	Self-reported non-specific psychological distress	K10	34,3327; 406 (municipalities) 7803 (neighbourhoods)	Multi-level linear regression	Age, gender, ethnicity, marital status, education, household income	Deprivation/income, ethnic composition, population density	Complex patterns of associations dependent on level examining, whether covariates included etc., with both positive & negative associations—see paper	4
Fan et al. [89]	2011–2015 (2013)	China	Community, City	6 million (city), 4000 (community)	GINI (income)	Symptoms of depression	CES-D-10	6540/8414; 450 (community), 116 (city)	Multilevel linear regression	Age, gender, marital status, socioeconomic status, physical health, lifestyle habits, chronic disease, physical disability, Body Mass Index (BMI)	Public health investment, community infrastructure, community elderly activity centre	Association between higher city-level inequality and depression (coefficient = 2.88, p < 0.01), which disappears after controlling for public health investment. Former effect only present in the 'non-poor' group	3
Fernandez-Nino et al. [97]	2012 (2010)	Mexico	Locality, municipality, state	45,616 (municipality)	GINI (income)	Caseness for depression	CES-D	7867; 2456	Multi-level logistic regression	Age, sex, civil status, education, paid job, participation in household decision making, illnesses, activities of daily living, instrumental activities, history of physical violence, accident incidence, household assets	Municipality and state deprivation	No association at the municipality (OR = 1.68, *p* > 0.1) or state (OR = 0.45, *p* > 0.1) level	4
Fiscella and Franks [54]	1982–1987 (1971–1975)	US	Primary sampling unit	NA	Proportion of total income earned by the poorest 50%	Symptoms of depression	Subscale of the general well-being schedule (GWB)	6913; 105	Multi-level linear regression	Age, sex, household income	None	Association between higher inequality and depression (β = −0.21, *p* < 0.05)	3
Fone et al. [98]	2003–2010 (2001)	Wales	Lower layer super output area (LSOA), unitary authority (A)	134,271	GINI (income)	General mental health symptoms (& caseness)	MH component of the SF-36	88,623; 1887 (LSOA), 22 (UA)	Multi-level linear and logistic regression	Age, sex, education, employment, housing tenure, household socioeconomic level	Deprivation	Association between higher inequality and better mental health at LSOA level (low deprivation areas only) (β = 0.7, *p* = 0.04); association between higher inequality and poorer mental health at UA level (β = −1.35, *p* = 0.01)	2
Fujita et al. [108]	2012–2016 (2013)	Japan	District and household	58,480	GINI (income)	Three-year incidence of a mood disorder	Diagnosed mood disorder according to ICD-10 categories F30-F39	116,658; 492 (districts), 83,594 (households)	Multi-level logistic regression	Age, sex, household type, equivalent income	Number of residents, number of institutions, average income	No association (OR = 1, *p* = 1)	4
Gresenz et al. [99]	1997–1998 (1990, 1996–1997)	US	State, Community	NA (community), 5 million (state)	GINI (income), Robin Hood index, share of total income earned by 50% of families with lowest income	Caseness for anxiety or depression disorder; general mental health symptoms	MH component of the SF-36; WMH-CIDI	6925; 60 (community), NA (state)	Multi-level linear and logistic regression	Age, race, gender, number of family members, family income	Income	No association at community (β = −0.45, *p* > 0.1) or state (β = 1.27, *p* > 0.1) level	4
Haithcoat et al. [91]	2014–2016 (2016)	US	State	6 million	GINI (income)	Self-reported depression diagnosis	Self-report	954,671; 48	Multi-level logistic regression	Age, gender, ethnicity, education, income, relationship status, health insurance, smoker or not, recent alcohol use, recent exercise history	Median income, percentage of households receiving Supplemental Nutrition Assistance Program (SNAP) benefits, percentage of non-institutionalized adults who have health insurance	Association between higher income inequality and lower odds of depression (OR = 0.01, *p* < 0.05)	3
Hanandita and Tampubolon [73]	2007 (2007)	Indonesia	District	1471	GINI (income)	General mental health symptoms (& caseness)	20-item Self-Reporting Questionnaire (SRQ)	57,7548; 440	Linear, poisson and probit regression	Age, sex, marital status, education, employment, physical activity, frequent smoker, heavy drinker, chronic illness, household size, household urbanicity, per capita household expenditure	Deprivation	Association between higher inequality and poorer general mental health (β = 3.59, *p* < 0.01)	3
Henderson et al. [100]	1991–1992 (1990)	US	State	5 million	GINI (income)	Symptoms of depression (& caseness)	AUDADIS	42,862; 48	Logistic regression	Age, ethnicity, education, household family size, urbanicity, household income	Income	No association for males (OR = 0.9, *p* > 0.05) or females (OR = 1.09, *p* > 0.05)	3
Kahn et al. [74]	1990 (1991)	US	State	5 million	GINI (income)	Caseness for depression	CES-D	8,060; 50	Logistic regression	Age, marital status, education, ethnicity, household population, household income	None	Association between higher inequality and depressive symptoms (OR = 1.3, *p* < 0.05), particularly amongst the poorest women	2
Kirkbride et al. [75]	1996–2000 (2004)	UK	Statistical ward	6195	GINI (income)	Psychosis incidence	SCAN	427; 56	Multi-level Bayesian modelling	Age, sex, ethnicity, socioeconomic level	Deprivation, population density, social fragmentation index, social cohesion	Association between higher inequality and non-affective psychosis (RR = 1.25, *p* < 0.05) but not affective psychosis	4
Lee and Park [101]	2009 (2009)	Korea	Community	402,084	GINI (income)	Caseness for depression	CES-D	230,715; 253	Multi-level logistic regression	Age, sex, education, number of illnesses, living alone, family income	Community mean income	No association (OR = 0.87, *p* > 0.05)	4
Lin et al. [84]	2014 (2014)	China	City	6,681,156	GINI (income)	Self-reported non-specific psychological distress	K6	15,999; 8	Multi-level linear regression and Spearman rank correlation	Age, gender, education, category of ‘Hokuo’ (resident status), marital status, years of residence, dimensions of 'social integration' defined by PCA (social insurance, social communication, acculturation and integration will, socioeconomic status)	None	Gini coefficient correlated with distress (RS = −0.04, *p* < 0.001), but not significant predictor in regression analyses with covariates added (β = 0.08, *p* > 0.05)	2
Marshall et al. [90]	2002–2003 (2003–2004)	England	Middle superior output area (MSOA)	7200	GINI (house prices)	Caseness for depression	CES-D	10,644; 2000 +	Multi-level logistic regression	Age, sex, ethnicity, education, household wealth, economic activity, living arrangements	Wealth, deprivation	Association between higher inequality and lower levels of depression (OR = 0.81, *p* < 0.05) that was strongest for the poorest individuals	4
Matthew and Brodersen [92]	2006–2014 (2006–2014)	US	State	6 million	GINI (income)	Self-reported diagnosis of depression or anxiety, self-reported 30-day incidence of mental health problems	Single item self-report questions	2,859,683; 48	Multi-level binary probit regression	Age, sex, ethnicity, marital status, income, health insurance status, education level, household size, employment status	Median household income	Higher inequality associated with lower likelihood of depression (−0.08, *p* < 0.01) and mental health problems (−0.02, *p* < 0.05), but not anxiety (−0.01, *p* > 0.05), with a stronger effect amongst low-income participants	3
Messias et al. [76]	2006–2008 (2006)	US	State	5.5 million	GINI (income)	Caseness for depression	PHQ-8	235,067; 45	Linear regression	None	Income, inequality, percentage with a college degree, percentage over 65	Association between higher inequality and depression (unstandardized beta = 43.67, p < 0.001)	2
Muramatsu [77]	1993–1994 (1990)	US	County	150,000	GINI (income)	Symptoms of depression	CES-D	6640; 211	Multi-level linear regression	Age, gender, education, family income, family net assets, marital status, physical health, ethnicity	Income	Association between higher inequality and lower depression (β = 2.59, *p* < 0.01)	4
Pabayo et al. [78]	2001–2005 (2000)	US	State	5.5 million	GINI (income)	Incidence of depression	AUDADIS	34,653; 50	Multi-level logistic regression	Age, sex, ethnicity, education, marital status, personal / family history of depression, past-year life events, household income, health	Income, proportion in poverty, proportion African–American, population size, census division	Association between higher inequality and depression for women (OR = 1.5, *p* < 0.05) but not for men	4
Pabayo et al. [85]	2001–2005 (2000)	US	State (and the District of Columbia)	5.5 million	GINI (income)	Presence of a PTSD episode in three-year follow-up (incident/persistent/recurrent)	AUDADIS	27,503; 51	Multi-level logistic regression	Age, sex, ethnicity, education, marital status, household income, years since experienced PTSD, urbanicity	Median income, proportion in poverty, proportion African–American, population size, census division	High inequality was associated with three-year PTSD incidence (OR = 1.3, CIs = 1.04–1.63) but not recurrence/persistence (OR = 1.02, CIs = 0.85–1.22)	4
Peterson et al. [102]	1998 (1998)	US	County	150,000	GINI (income)	Mental health symptoms	MH component of the SF-12	16,261; 88	Multi-level linear regression	Age, gender, race/ethnicity, level of educational attainment, lack of health insurance prior year, whether adjusted household income was < 200% of the federal poverty level, absence of a usual source of medical care, lack of social support, lack of employment outside the time for pay. self-assessed general health status, physical component of the SF-12, lack of leisure time exercise, current smoking status	Availability of primary care physicians, psychiatrists, inpatient psychiatric beds, presence/absence of hospital-based psychiatric or social work services, number violent crimes, proportion of county residents living in poverty, proportion unemployed, median household income, proportion adults 25 or older with high school degree or equivalent, violent crimes, female-headed households, proportion vacant housing, Two components of the Comprehensive Social Capital Index, rural / urban status	No association between inequality and SF-12 scores (coefficients = −0.01 to 0.01)	4
Sebastian et al. [93]	2014 (2014)	Sweden	Municipality	19,956	GINI (income)	Self-reported non-specific psychological distress	GHQ-12	21,004; 32	Single-level log-binomial regression analysis	Age, sex, education, civil status, immigration background, occupation, income level, relative income	Average income in each municipality, type of municipality	Individuals from municipalities with intermediate inequality (only) showed lower psychological distress than those from the municipality with the lowest inequality (PR = 0.89, CIs = 0.79–1; PR = 0.87 CIs = 0.75–0.99)	3
Sommet et al. [86]	1999–2013 (1999–2013)	Switzerland	Municipality	5570	GINI (income)	Self-reported frequency of “negative feelings”	Single-item question	14,790; 1745	Multi-level linear regression	Age, sex, education, employment, income	Total population, poverty, unemployment, income per capita	(Within-individual) high inequality associated with greater psychological distress, but only for those facing 'financial scarcity' (β = 2.82, *p* = 0.002)	3
Sturm and Gresenz [103]	1997–1998 (1990)	US	Metropolitan area or economic area	NA	GINI (income)	Caseness for depression or anxiety disorder	WMH-CIDI (short-form)	8,235; 60	Logistic regression	Age, sex, ethnicity, education, family size, family income	None	No association (*p* > 0.1)	2
Tibber et al. [94]	1998–2006 (2001)	England	Census Area Statistics Ward	10,795	GINI (deprivation)	Positive, Negative, Disorganised symptom dimension scores	SAPS, SANS	319; 113	Multi-level linear regression	Age, gender, socioeconomic status, other symptom scores	Population density, deprivation, social fragmentation, social capital, ethnic density, ethnic segregation	Higher inequality associated with lower negative symptoms only (coefficient = −2.06, *p* < 0.01)	4
Weich et al. [104]	1991 (1991)	Britain	Region	3 million	Gini (income); the mean log deviation; Theil index; half the squared coefficient of variation	Caseness for general mental health	GHQ	8191; 18	Logistic regression	Age, sex, ethnicity, employment, social class, physical health problems, housing tenure, household income, marital status, education	Income	Association between higher inequality and poorer MH in wealthier participants (OR = 1.31, *p* = 0.02); higher inequality and better MH in poorer participants (OR = 0.42, *p* < 0.001)	2

Key measures include: years over which data were gathered (inequality data year in brackets), mental health (MH) variable/s, sample size (individual level; higher-order level), quality index (Qi)

*MH*  mental health; *NA* data not available; *OR* odds ratio; *IRR*  incident rate ratio; *SF-36*  Short Form Health Survey; *OCCPI*  Operational Criteria Checklist for Psychotic Illness; *WMH-CIDI*  Composite International Diagnostic Interview; *CES-D*  Centre for Epidemiological Studies Depression Scale; *WHOQOL-BREF * Mental health component of the World Health Organization Quality of Life Assessment; *AUDADIS*  Alcohol Use Disorder and Associated Disabilities Interview Schedule; *K6/K10*  Kessler Psychological Distress Scale; *SAPS * Scale for the Assessment of Positive Symptoms; *SANS * Scale for the Assessment of Negative Symptoms; *GHQ*  General Health Questionnaire; *PHQ*  Patient Health Questionnaire; *SCAN*  Clinical Assessment in Neuropsychiatry

### Quality assessment

Following the approach of Uphoff and colleagues [65], studies were scored for *quality* rather than *risk of bias*, as appropriate for a critical appraisal of large-scale cross-sectional and/or ecological data. The following criteria were used to create a Quality Index (Qi): (i) validity of key measures, (ii) sample size, (iii) inclusion of appropriate confounder variables, and (iv) optimal statistical analyses. Items (i) and (ii) were taken directly from Uphoff and colleagues [65], and (iii) and (iv) were custom-developed to afford a more stringent assessment of quality in line with the research question; thus, multi-level analyses that control for absolute deprivation were deemed necessary for a convincing association to be demonstrated between inequality and mental health. See Supplementary Information 2 for further details.

### Data synthesis

A vote-count approach was used to identify the proportion of studies that were consistent with: (a) the IIH, (b) the MNH, or (c) neither (i.e. no association between inequality and mental health). Note: we use the term ‘consistent with’ since without an established direction of causality and elucidation of mediating mechanisms, associations between inequality and mental health do not definitively *prove* the IIH *or* the MNH. Following Wilkinson and Pickett’s [56], supportive categories were further broken down into sub-categories of ‘wholly supportive’ (where *only* significant associations were found in the defined direction), and ‘partially supportive’ (where some significant association in the defined direction and some null findings were reported). Missing data were excluded from syntheses rather than assumptions being made.

In addition, we undertook several ‘sub-analyses’, with the same vote-count approach implemented on a subset of studies. First, to assess the scale invariance of any reported effects, findings were explored at different geographical scales. Since the scale at which to stratify studies is relatively arbitrary, we took two principled approaches. Data were stratified according to mean population size of the geographical region of interest, with strata (< 45,000, ≥ 45,000, ≥ 4 million) defined *post hoc* to generate approximately equal sized groups. Data were also stratified following a system used previously [56], with studies identified as focusing on regions of interest that corresponded broadly to: (i) states, regions and cities, and (ii) counties, tracts and parishes (Table 2). These corresponded to studies with regions of interest with mean population sizes that ranged from ~ 1500–190,000 and ~ 290,000–6 million.

**Table 2:** Support for the income inequality and mixed neighbourhood hypotheses

		Wholly supportive of the IIH	Partially supportive of the IIH	Unsupportive of either	Partially supportive of the MNH	Wholly supportive of the MNH	Total	Supportive of the IIH	Supportive of the MNH
(i) All studies		8 (19.05)	15 (35.71)	14 (33.33)	3 (7.14)	2 (4.76)	42	23 (54.76)	5 (11.9)
(ii) Higher quality studies		1 (6.25)	6 (37.5)	7 (43.75)	1 (6.25)	1 (6.25)	16	7 (43.75)	2 (12.5)
(iii) Controlled for absolute deprivation	At lower-level	6 (17.14)	11 (31.43)	13 (37.14)	3 (8.57)	2 (5.71)	35	17 (48.57)	5 (14.29)
	At higher-level	3 (10)	10 (33.33)	12 (40)	3 (10)	2 (6.67)	30	13 (43.33)	5 (16.67)
	At both levels	2 (7.69)	8 (30.77)	11 (42.31)	3 (11.54)	2 (7.69)	26	10 (38.46)	5 (19.23)
(iv) Stratified by region mean pop size	< 45,000	1 (7.69)	6 (46.15)	3 (23.08)	2 (15.38)	1 (7.69)	13	7 (53.85)	3 (23.08)
	≥ 45,000	3 (23.08)	3 (23.08)	7 (53.85)	0 (0)	0 (0)	13	6 (46.15)	0 (0)
	≥ 4 million	3 (23.08)	6 (46.15)	2 (15.38)	1 (7.69)	1 (7.69)	13	9 (69.23)	2 (15.38)
(v) Stratified by region type	Counties, tracts, parishes (or similar)	3 (14.29)	8 (38.1)	7 (33.33)	2 (9.52)	1 (4.76)	21	11 (52.38)	3 (14.29)
	States, regions, cities (or similar)	4 (22.22)	7 (38.89)	5 (27.78)	1 (5.56)	1 (5.56)	18	11 (61.11)	2 (11.11)
(vi) Stratified by mental health condition	General mental health	2 (11.76)	5 (29.41)	8 (47.06)	2 (11.76)	0 (0)	17	7 (41.18)	2 (11.76)
	Depression	5 (26.32)	6 (31.58)	6 (31.58)	0 (0)	2 (10.53)	19	11 (57.89)	2 (10.53)
	Psychosis	1 (20)	3 (60)	0 (0)	1 (20)	0 (0)	5	4 (80)	1 (20)
(vii) Stratified by economic status of country	LMIC	4 (36.36)	4 (36.36)	3 (27.27)	0 (0)	0 (0)	11	8 (72.72)	0 (0)
	HIC	4 (12.9)	11 (35.48)	11 (35.48)	3 (9.68)	2 (6.45)	31	15 (48.39)	5 (16.13)

The number of studies that were supportive of the Income Inequality Hypothesis (IIH), supportive of the Mixed Neighbourhood Hypothesis (MNH), or else unsupportive of either theory, are presented for: (i) all studies, (ii) higher quality studies only (i.e. those obtaining a maximum score of four on the Quality Index), (iii) studies that controlled for absolute deprivation only (at the lower-level, higher-level and both), (iv) studies stratified by the mean population size of the geographical area of interest (*X* < 45,000; 45,000 $\le$ *X*  <  4 million; *X*  ≥  4million), (v) studies stratified by region type, (vi) studies stratified by mental health presentation, and (vii) studies stratified by economic status of country from which the data were gathered. For these data, percentages of total studies (row total) are also presented in brackets. In the final two columns partially and wholly supportive data are collapsed for ease of interpretation

*LMIC*  low or medium income countries; *HIC* high income countries

Second, to determine whether study quality introduced any bias in findings, findings were also explored for higher quality studies only, i.e. those scoring four on the quality index (Qi). Third, to test for the potentially confounding role of *absolute* deprivation, findings were explored in a subset of studies for which deprivation was controlled at the lower level (e.g. individual or household), higher level (e.g. state or county), and at both levels. Fourth, to determine whether patterns of association differed between mental health conditions, findings were also explored for studies involving different (primary) mental health conditions. Finally, in two further unplanned/*post hoc* analyses we also explored: (i) where interactions between inequality and absolute deprivation were reported, whether these selectively or disproportionately impacted negatively on the poor or the wealthy, and (ii) whether any findings reported held for low/medium (LMIC) and high income (HIC) countries, as defined by the World Bank Classification system [66].

## Results

A total of 1251 studies were initially identified; 42 of these met criteria for inclusion (Fig. 1). Table 1 presents studies that were retained along with key coded variables. This represented data from 7,744,469 participants residing in 110,247 geographical regions. The largest proportion of studies (*n* = 17, 40.48%) involved data gathered in the US. With respect to the mental health conditions examined, 19 (45.24%) investigated depression, 17 (40.48%) general mental health, 5 psychosis (11.9%) and 1 (2.38%) post-traumatic stress disorder (Table 1). The most common measure of inequality used was the Gini coefficient (*n* = 34, 80.95%), with four (9.52%) using multiple indices and four including single alternative indices.

### Findings based on all included studies

Considering all studies first, 54.76% (*n* = 23) were partially or wholly supportive of the IIH [67–89], whereas only 11.9% (*n* = 5) of studies were supportive of the MNH [90–94] (Table 2). In contrast, 33.33% (*n* = 14) of the studies were unsupportive of *either* hypothesis [95–108], three of which (21.43%) showed mixed findings [98, 104, 107] and the remaining 11 (78.57%) reporting only null findings*.*

Of 15 studies that were only *partially* supportive of the IIH, reasons for this included associations *only* being seen: in low-income participants or deprived wards [67, 68, 86], with respect to certain symptoms or presentations [70, 71, 75, 81, 85], prior to adjustment for covariates [84, 88, 89], in women [78], at the provincial but not county level [87], at a given time-lag [79]. Finally, one study found that inequality predicted variance in depression symptoms *between* but not *within* twin pairs [82].

Of three studies that were only *partially* supportive of MNH, reasons for this included associations *only* being seen with respect to a subset of psychosis symptoms [94] or mental health presentations [92]. Finally, one study found that individuals from municipalities with intermediate (but not high) inequality reported lower psychological distress than participants from municipalities with the lowest inequality [93].

Of the three studies that were found to be unsupportive of either hypothesis due to mixed findings, reasons for this included that the sign/nature of the association depended on: the level of neighbourhood deprivation and geographical scale of analysis [98], the wealth of participants [104], or the level of analysis/choice of covariates included [107].

### Quality indices and the impact of study quality

Of the 42 studies included, 5 were deemed to have invalid measure/s (11.9%), 6 had an inadequate sample size (14.29%), 16 failed to control for *absolute* deprivation (38.1%) and 12 used non-optimal analyses (28.57%). The main finding (described above), however, was preserved in the 16 highest quality studies (Qi = 4) (Table 2), although the pattern was slightly less pronounced: 43.75% supported the IIH [67, 75, 77, 78, 85, 87, 88] and 12.5% supported the MNH [90, 94].

### Impact of absolute deprivation as a covariate

A similar pattern emerged when we restricted analyses to studies that controlled for absolute levels of deprivation, at either lower-order, higher-order, or both levels (Table 2). Twenty-six studies controlled for absolute deprivation at both levels, with twice as many studies supporting the IIH (*n* = 10, 40%) [67, 73, 75, 77, 78, 81, 85–88] compared with the MNH (*n* = 5, 20%) [90–94].

### Effects of geographical scale

There was little to suggest that the association between inequality and mental health was *dependent* on geographical scale, irrespective of whether this was defined by region mean population size or region *type*. Thus, across these analyses 46.15–69.23% of studies supported the IIH whereas only 0–23.08% of studies supported the MNH. It is worth noting, however, that in both sets of analyses the highest support for the IIH was found at the largest geographical scale.

### Patterns for different mental health conditions

There was stronger support for the IIH than there was for the MNH, across all mental health categories examined: *general* mental health (41.18% vs. 11.76%), depression (57.89% vs. 10.53%) and psychosis (80% vs. 20%), although the pattern was most pronounced for psychosis.

### Role of absolute deprivation

Twenty of the 42 studies included tested for interactions between inequality and *absolute* deprivation, either by adding cross-level interaction terms or stratification of data by indices of deprivation or wealth. Of these, 14 found evidence of an interaction. Eight of these indicated that the *poor fared worse*; i.e. where associations between *higher* inequality and *poorer* mental health were reported these were more pronounced amongst the deprived, or where associations between *higher* inequality and *better* mental health were reported, these were specific to wealthy areas [67, 68, 74, 80, 83, 84, 86, 98]. Conversely, six indicated that the *rich fared worse*, such that they were linked to more positive and/or less negative effects of inequality [87, 89, 90, 92, 104, 107].

### Effects of country-level economic status

Eleven studies included data from LMICs and 31 included data from HICs. Whilst both showed higher support for the IIH than the MNH, the pattern was much more pronounced in the LMICs (72.72% vs. 0%) than in the HICs (48.39% vs. 16.13%).

## Discussion

Based on a systematic review of the literature we consistently found greater support for the IIH over the MNH. This pattern was not dependent on study quality, spatial scale, adjustment for absolute deprivation, nor country income level. However, a high proportion of studies supported *neither* hypothesis, reporting no significant association between inequality and mental health, or else mixed patterns of associations. To explain such a high level of null findings one might posit two possible explanations. First, that findings supportive of the IIH have arisen purely by chance, but are over-represented in the literature [109, 110]. Second, that the association is real, but statistically small and/or potentially dependent on other moderating variables. Consistent with the latter interpretation, a parallel modest association has also been documented between higher inequality and poorer *physical* health [27], with overlapping mechanisms having been proposed for mental and physical health [31]. Nonetheless, in reviewing the extant literature we identified a number of limitations, most notably a lack of adequate control for absolute deprivation (at the lower and higher-order levels) and the use of suboptimal (i.e. single-level) analyses.

Considering more specific predictions of the IIH, the findings reported are broadly consistent with the notion that the effects of inequality are not limited to poorer members of society [11]. The association between higher inequality and poorer mental health persisted after controlling for absolute deprivation and was evidenced in HICs *and* LMICs. In addition, where studies investigated an interaction between inequality and absolute deprivation, a roughly equal proportion indicated that the poor or the rich were negatively impacted. Assuming a casual association (more on this below), this is a crucial finding with implications for the potential scale of impact and ways of incentivising change, since it implies that *all* segments of society stand to be affected by the negative effects of inequality, and by inference, stand to gain by addressing the issue.

With respect to geographical scale [55, 96], the reported association persisted across all spatial scales studied, although it was somewhat more pronounced at higher spatial scales. Drawing on the SAH, these findings are consistent with social comparison [111] and social rank [112] theories, which posit that the negative effects of social comparisons operate across multiple reference groups and spatial scales, including the local [113, 114]. Such scaling effects may also be supported by the growing ubiquity of social/digital tools such as social network sites [115], which have arguably transformed the potential scope and scale of such comparative processes [116].

Whilst the IIH makes no explicit predictions about the specificity of effects on different mental health conditions, stratification by mental health suggested that the association between inequality and mental health may be particularly pronounced in psychosis (although the sample size of studies was very small). It is unclear why this might be the case; however, one tentative hypothesis is that the lack of social integration and trust that arguably characterises unequal communities (according to the SCH and SAH) may be particularly conducive to experiences of paranoia, a core symptom of psychosis [117]. These findings, if found to hold with further research, have potential implications for the commissioning and delivery of psychosis services (more on this below).

With respect to the limitations of this review, no measure of sampling bias was included. Some studies used convenience sampling, and others purposely over-sampled specific ethnic groups or geographical regions so that conclusions could be drawn about low incidence groups (see Supplementary Information 3). Nonetheless, this may limit the generalizability of findings. Further, whilst the decision was based on firm theoretical grounds [60–62], the lack of integration of effect sizes across studies means that the real-world significance of the findings are difficult to gauge. Finally, no conclusions can be drawn about the direction of causality or underlying mechanisms. Whilst these were not the foci of the review, in the absence of such information the findings we report are merely *consistent* with the IIH. Nonetheless, it is worth noting that in a review of the literature into the association between inequality and health (more generally), the authors concluded that there was good support for the main criteria used to test for causality within a causal epidemiological framework, i.e. temporality, biological plausibility, consistency and lack of alternative explanations [118].

If we accept the proposed notion of a *casual* association between inequality and mental health, several important implications emerge from our findings. Most fundamentally, they suggest that rising levels of inequality may drive increases in the incidence of mental health disorders, and arguably as a consequence, that inequality (alongside poverty and other environmental factors) should be placed at the centre of psychiatry and applied psychology [5]. For example, national guidelines for Early Intervention Psychosis services in the UK [119] state that commissioning “should be underpinned by estimated local incidence of psychosis, derived to incorporate a range of demographic features such as ethnicity, age, population density and deprivation” (p. 6), and to this we would add inequality as a further important factor for consideration.

The findings also raise the possibility that national health expenditure, which has traditionally focused on the development and provision of mental health services that work with the individual to target symptom reduction [120], may need to include parallel investments into a wider range of services as part of a more systemic, preventative approach if they are to be effective [121, 122]. For example, Marmot [123] has argued for the importance of focusing on “early child development and education, work environments, building healthy communities and supporting active social engagement of older people” in overcoming the effects of social inequality on health (p. 153). Conversely, we would suggest that the findings strongly call into question the wisdom of implementing mixed tenure policies that aim to create mixed communities, including with respect to income [124].

Relatedly, an argument might also be made for tackling inequality more directly, i.e. as primary causal/upstream factor, as part of government policy. Thus, many academics, including economists [125] and epidemiologists [123], have argued that trends for rising inequality can be reversed through targeted changes in social policy without sacrificing overall economic growth [126]. Proven tools in this regard include progressive taxation and focused expenditure aimed at improving education and reducing hunger and poverty [127, 128]. Relatedly, our finding that LMICs may be *particularly* susceptible to the negative effects of income inequality, suggests that international development and aid programmes, which have traditionally focused on increasing economic *growth*, may benefit from a broader remit that includes working to reduce economic *inequality* [129], a perspective that is reflected in the UN Sustainable Development Goals (Goal 10: *‘Reduce inequality within and among countries’*, p.14) [130].

## Conclusions

This systematic review highlights an association between higher levels of income inequality and poorer adult mental health at the subnational level. Whilst the review did not attempt to identify the mechanisms or direction of this association, the conclusions drawn reinforce the importance of inequality in potentially contributing to mental health problems in the population. Further research into the causal strength of such environmental predictors on psychological distress is urgently required so we can assess the potential value of implementing interventions to ameliorate the negative effects of inequality. This research effort now needs to gather pace, and is we would argue, underpinned by an ethical imperative. In a recent report entitled ‘Britain in the 2020s’ the Institute for Public Policy Research [131] predicted that inequality will “surge” over the course of the decade (p. 12), with the income of the rich forecasted to rise 11 times faster than the incomes of the poor, and an extra 3.6 million predicted to fall into poverty within this time-frame.

## Supplementary Information

Below is the link to the electronic supplementary material.

Supplementary file1 (DOCX 84 KB)

## Data Availability

Not applicable.
