# Supplementary material for: The association between income inequality and adult mental health at the subnational level—a systematic review
Source: Soc Psychiatry Psychiatr Epidemiol. 2021 Aug 13;57(1):1–24. doi: 10.1007/s00127-021-02159-w (PMC8761134; doi:10.1007/s00127-021-02159-w)
Supplement: Supplementary file 1 — Supplementary file1 (DOCX 84 KB) [file 127_2021_2159_MOESM1_ESM.docx]

**Supplementary Information 1 – Search Terms**

Synonyms for income inequality and specific indices of income inequality were derived from a review paper of income inequality measures [1]. Relevant synonyms for mental health difficulties / conditions (e.g. ‘psychological disorder’, ‘mental illness’) and diagnostic categories (e.g. ‘schizophrenia’, ‘anxiety’, ‘depression’) were derived from multiple sources including: previous relevant meta-analyses [2], published diagnostic classification systems [3–5] and diagnostic terms used in the Improving Access to Psychological Therapies (IAPT) and IAPT for Severe Mental Illness services [6]. This generated 11 search terms for income inequality (combined using the OR operator) and 52 search terms for mental health / diagnostic categories (combined using the OR operator), which were in turn combined using the AND operator. These are presented below.

[“income inequality” OR “relative deprivation” OR “gini coefficient” OR “generalised entropy” OR “Atkinson index” OR “decile ratio” OR “kakwani progressivity” OR “proportion of total income earned” OR “Robin Hood index” OR “pietra ratio” OR “sen poverty measure”]

AND

[“mental disorder” OR “mental illness” OR “mental health” OR “psychological disorder” OR “personality disorder” OR “schizophrenia” OR “schizophrenic” OR “psychosis” OR “psychotic” OR ”bipolar” OR “schizoaffective” OR “manic depression” OR “affective disorder” OR “depression” OR “anxiety” OR “PTSD” OR “post traumatic stress disorder” OR “eating disorder” OR “social phobia” OR “social anxiety” OR “panic” OR “generalised anxiety disorder” OR “GAD” OR “obsessive compulsive disorder” OR “OCD” OR “psychological disturbance” OR “emotional problem” OR “schizophreniform” OR “separation anxiety” OR “agoraphobia” OR “body dysmorphic” OR “body dysmorphia” OR “bulimia” OR “binge-eating” OR “hypomania” OR “mania” OR “dysthymia” OR “cyclothymia” OR “paranoia“ OR “paranoid“ OR “delusional“ OR “schizotypal“ OR “schizoid“ OR “dissocial“ OR “emotionally unstable“ OR “histrionic“ OR “anankastic“ OR “avoidant“ OR “dependent personality disorder” OR “dissociative“ OR “antisocial“ OR “borderline“].

**Supplementary Information 2 – Quality Index**

Q1 - validity of key measures: measures were deemed valid if they accurately mapped onto the research question and had been validated in previous peer-reviewed research. A score of one was given if *both* key measures (i.e. inequality and the mental health outcome under study) were deemed valid.

Q2 - sample size: for a single-level regression analysis or partial correlation the sample size was deemed appropriate (score of one) if it met the ‘one in ten’ heuristic, which defines a minimum sample size of ten observations per predictor variable [7, 8]. For multi-level analyses, an additional criteria of a minimum of 30 units, e.g. states or neighbourhoods, at the higher level also had to be met [9]. A score of one was given if *both* these criteria were met.

Q3 - inclusion of appropriate confounder variables: studies were given a score of one if analyses controlled for *absolute* deprivation at the individual / household level *as well as* at the superordinate level (e.g. neighbourhood).

Q4 - optimal statistical analyses: A score of one was given if multi-level analyses were used, with data included at an individual level as well as at a higher order level.

Supplementary Information 3 – Additional information on studies included in the review

**Supplementary Table - Studies included in the review – additional information.** The full list of studies included in this review is presented along with a number of additional variables not included in the main body of the thesis: population sample, age, male-to-female ratio, quality index item scores for Q1 (validity of variables), Q2 (adequate sample size), Q3 (appropriate control variables), Q4 (optimal analyses), and Qi (quality index total, i.e. the sum of Q1-4), results code [-1=wholly supportive of the Mixed Neighbourhood Hypothesis (MNH), -1*=partially supportive of the MNH, 0=supportive of neither hypothesis, 1*=partially supportive of the Income Inequality Hypothesis (IIH), 1=wholly supportive of the IIH], method of data collection, sampling strategy. NA=data not available.

| **Study** | **Population Sample** | **Age** | **Male-to -female ratio** | **Q1** | **Q2** | **Q3** | **Q4** | **Qi** | **Results Code** | **Method of data collection** | **Sampling strategy** |
| --- | --- | --- | --- | --- | --- | --- | --- | --- | --- | --- | --- |
| Ahern & Galea (2006) | Post-911 New York adults | >=18 | 0.78 | 1 | 1 | 1 | 1 | 4 | 1* | Phone-based interview | Probability sample (random dial; stratified by geography); over-sampling of World Trade Centre (WTC) site |
| Adjaye-Gbewonyo et al. (2016) | Adults | ≥15 | 0.55 | 1 | 1 | 1 | 1 | 4 | 0 | Questionnaires | Multi-stage stratified |
| Bechtel et al. (2012) | Adults | >=15 | NA | 1 | 1 | 0 | 0 | 2 | 0 | Face-to-face interview | Probability sample (stratified by geography); under-sampling of remote areas |
| Bisung et al. (2018) | Adult women | ≥18 | 0 | 0 | 0 | 1 | 1 | 2 | 0 | Face-to-face interview | Probability sample (stratified by area socioeconomic status and age); over-sampling of older women |
| Bocoum et al. (2019) | Teenagers and adults | ≥12 | NA | 0 | 1 | 0 | 0 | 1 | 1* | Face-to-face interview | Probability sample (multi-stage stratified); over-sampling of the young and elderly |
| Boydell et al. (2004) | First Episode Psychosis | NA | 1.31 | 1 | 0 | 0 | 1 | 2 | 1* | Review of clinical records | Convenience sample (all incident cases presenting to services) |
| Burns & Esterhuizen (2008) | First Episode Psychosis | 15-49 | 2.4 | 1 | 0 | 0 | 0 | 1 | 1 | Review of clinical records | Convenience sample (all incident cases presenting to services) |
| Burns et al. (2017) | Teenagers and adults | ≥15 | 0.67 | 1 | 1 | 0 | 1 | 3 | 1 | Face-to-face interview | Probability sample (multi-stage stratified) |
| Chen et al. (2019) | Adults | ≥18 | 0.9 | 1 | 1 | 1 | 0 | 3 | 1* | Face-to-face interview | Probability sample (multi-stage stratified); over-sampling of certain ethnicities |
| Chiavegatto Filho et al. (2013) | Adults | ≥18 | 0.79 | 1 | 1 | 0 | 1 | 3 | 1* | Face-to-face interviews | Multi-stage stratified |
| Choi et al. (2015) | Older adults | >50 | 0.69 | 1 | 1 | 0 | 0 | 2 | 1* | Face-to-face and phone-based interviews | Multi-stage stratified; oversampling of certain ethnic groups |
| Cohen-Cline et al. (2018) | Adult same-sex twins raised together | ≥18 | 0.53 | 1 | 1 | 0 | 1 | 3 | 1* | Postal Questionnaire | Non-probability sample (participants contacted or self-select) |
| Dev & Kim (2020) | Adults | ≥50 | 1.11 | 1 | 1 | 1 | 1 | 4 | 1* | Face-to-face and phone-based interview | Probability sample (multi-stage stratified); over-sampling of certain ethnicities |
| Ding et al. (2020) | Adults | ≥18 | 0.99 | 1 | 1 | 1 | 1 | 4 | 1* | Postal Questionnaire | Probability sample (multi-stage stratified) |
| Drukker et al. (2004) | Parents | 35-45 | NA | 1 | 1 | 0 | 1 | 3 | 0 | Distributed questionnaires | Probability sample |
| Du et al. (2019) | Adolescents and adults | ≥16 | 0.94 (T1) | 1 | 1 | 0 | 1 | 3 | 1 | Face-to-face and phone interview | Probability sample (multi-stage stratified) |
| Erdem et al. (2019) | Adults | ≥19 | 0.84 | 1 | 1 | 1 | 1 | 4 | 0 | Questionnaire survey | Probability sample |
| Fan et al. (2020) | Adults | ≥45 | 0.93 | 1 | 1 | 0 | 1 | 3 | 1* | Face-to-face computer-assisted interview | Probability sample (multi-stage stratified) |
| Fernandez-Nino et al. (2014) | Older adults | ≥60 | 0.87 | 1 | 1 | 1 | 1 | 4 | 0 | Face-to-face interview | Probability sample (stratified by geography) |
| Fiscella & Franks (2000) | Adults | 25-74 | NA | 1 | 1 | 0 | 1 | 3 | 1 | Face-to-face interview | Probability sample (stratified by geography and demographics); over-sampling of poor areas, women of child-bearing age & the elderly |
| Fone et al. (2013) | Adults | 18-74 | 0.87 | 1 | 0 | 1 | 1 | 3 | 0 | Face-to-face interview | Probability sample (stratified by geography) |
| Fujita et al. (2019) | Adults | ≥20 | 0.93 | 1 | 1 | 1 | 1 | 4 | 0 | Review of tax and insurance records | Convenience sample of National Health Insurance beneficiaries |
| Gresenz et al. (2001) | Adults | <65 | NA | 1 | 1 | 1 | 1 | 4 | 0 | Phone-based interview | Probability sample (stratified by geography and demographics); over-sampling of individuals with mental illness and/or low income |
| Haithcoat et al. (2019) | Adults | ≥18 | 0.82 | 0 | 1 | 1 | 1 | 3 | -1 | Phone-based interview | Probability sample (stratified) |
| Hanandita & Tampubolon (2014) | Adults | ≥15 | 0.93 | 1 | 1 | 1 | 0 | 3 | 1 | Face-to-face interview | Probability sample (stratified by geography) |
| Henderson et al. (2004) | Adults | ≥18 | 0.71 | 1 | 1 | 1 | 0 | 3 | 0 | Face-to-face interview | Probability sample (stratified by geography and demographics); over-sampling of black people and 18-29 year olds |
| Kahn et al. ( 2000) | Adult mothers | ≥15 | 0 | 1 | 1 | 0 | 0 | 2 | 1 | Phone-based interview and postal questionnaire | Probability sample (stratified by geography and demographics); over-sampling of mothers of black and low birth-weight infants |
| Kirkbride et al. (2014) | First Episode Psychosis | 18-64 | 1.54 | 1 | 1 | 1 | 1 | 4 | 1* | Face-to-face interview | Convenience sample (incident cases presenting to services) |
| Lee & Park (2015) | Adults | 19-60+ | 0.85 | 1 | 1 | 1 | 1 | 4 | 0 | Face-to-face interview | Probability sample (stratified by geography and demographics) |
| Lin et al. (2017) | Adult migrants | ≥15 | 1.22 | 1 | 0 | 0 | 1 | 2 | 1* | Face-to-face interview | Probability sample (multi-stage stratified) |
| Marshall et al. (2014) | Older adults | ≥50 | 0.83 | 1 | 1 | 1 | 1 | 4 | -1 | Face-to-face interview | Probability sample (stratified by geography) |
| Matthew & Brodersen (2018) | Adults | ≥18 | 0.71 | 0 | 1 | 1 | 1 | 3 | -1* | Phone-based interview | Probability sample (random dial; stratified by geography) |
| Messias et al. (2011) | Adults | ≥18 | 0.96 | 1 | 1 | 0 | 0 | 2 | 1 | Phone-based interview | Probability sample (random dial; stratified by geography) |
| Muramatsu (2003) | Older adults | 70-103 | 0.61 | 1 | 1 | 1 | 1 | 4 | 1 | Face-to-face and phone-based interview | Probability sample (stratified by geography and demographics); over-sampling of residents of Florida and minority groups |
| Pabayo et al (2014) | Adults | ≥18 | 0.72 | 1 | 1 | 1 | 1 | 4 | 1* | Face-to-face interview | Probability sample (stratified by geography) |
| Pabayo et al. (2017) | Adults | ≥18 | 0.74 | 1 | 1 | 1 | 1 | 4 | 1* | Face-to-face interviews | Probability sampling (stratified by geography and sociodemographics); oversampling of black and hispanic individuals |
| Peterson et al. (2009) | Adults | ≥18 | 0.92 | 1 | 1 | 1 | 1 | 4 | 0 | Phone-based interviews | Probability sample (random dial; stratified by geography); over-sampling by race and gender |
| San Sebastian et al. (2018) | Adults | ≥25 | 0.88 | 1 | 1 | 1 | 0 | 3 | -1* | Questionnaire survey (on- or off-line) | Probability sampling (stratified by geography) |
| Sommet et al. (2018) | Adolescents and adults | ≥14 | 0.87 | 0 | 1 | 1 | 1 | 3 | 1* | Phone-based interview | Probability sampling (stratified by geography) |
| Sturm & Gresenz (2002) | Adults | NA | NA | 1 | 1 | 0 | 0 | 2 | 0 | Phone-based interview | Probability sample (random dial; stratified by geography) |
| Tibber et al. (2019) | Adolescents and adults with First Episode Psychosis | ≥16 | 1.93 | 1 | 1 | 1 | 1 | 4 | -1* | Face-to-face interview and review of clinical records | Convenience sample (individuals presenting to services) |
| Weich et al. (2001) | Adults | 16-75 | NA | 1 | 0 | 1 | 0 | 2 | 0 | Face-to-face and phone-based interview | Probability sample (stratified by geography) |
|  |  |  |  |  |  |  |  |  |  |  |  |

References

1. De Maio FG (2007) Income inequality measures. J Epidemiol Community Health 61:849–52. https://doi.org/10.1136/jech.2006.052969

2. Barratt H, Rojas-García A, Clarke K, et al (2016) Epidemiology of Mental Health Attendances at Emergency Departments: Systematic Review and Meta-Analysis. PLoS One 11:e0154449. https://doi.org/10.1371/journal.pone.0154449

3. American Psychiatric Association (2000) Diagnostic and Statistical Manual of Mental Disorders - Fourth Edition, 4th ed. American Psychiatric Association, Washington, DC

4. American Psychiatric Association (2013) Diagnostic and Statistical Manual of Mental Disorders - Fifth Edition, 5th ed. American Psychiatric Association, Arlington, VA

5. World Health Organisation (1992) ICD-10 Classification of Mental and Behavioural Disorder: Clinical Descriptions and Diagnostic Guidelines. World Health Organisation, Geneva

6. Health and Social Care Information Centre (2014) Psychological Therapies, Annual Report on the use of IAPT services: England– 2013/14 EXPERIMENTAL STATISTICS

7. Austin PC, Steyerberg EW (2015) The number of subjects per variable required in linear regression analyses. J Clin Epidemiol 68:627–636. https://doi.org/10.1016/j.jclinepi.2014.12.014

8. Vittinghoff E, McCulloch CE (2007) Relaxing the rule of ten events per variable in logistic and Cox regression. Am J Epidemiol 165:710–8. https://doi.org/10.1093/aje/kwk052

9. Maas CJM, Hox JJ (2004) Robustness issues in multilevel regression analysis. Stat Neerl 58:127–137. https://doi.org/10.1046/j.0039-0402.2003.00252.x
